# Hyperkalemia Versus Pseudohyperkalemia Without ECG Changes in Acute Blast Crisis Progressing to Tumor Lysis Syndrome

**DOI:** 10.7759/cureus.81026

**Published:** 2025-03-23

**Authors:** Moiuz Chaudhri, Ayesha Samad, Jordan Lipschutz, Jose Iglesias

**Affiliations:** 1 Internal Medicine, Ocean University Medical Center, Brick Township, USA; 2 Nephrology, Ocean University Medical Center, Brick Township, USA; 3 Internal Medicine/Nephrology, Hackensack Meridian School of Medicine, Seton Hall University, Nutley, USA

**Keywords:** acute blast crisis, chronic myelomonocytic leukemia (cmml), ecg changes, hyperkalemia, pseudohyperkalemia, tumor lysis syndrome

## Abstract

Hyperkalemia is critical to recognize, but distinguishing it from pseudohyperkalemia is essential to avoid unnecessary treatment. We present a 66-year-old male with a history of myeloproliferative disorder and chronic myelomonocytic leukemia (CMML) who developed hyperleukocytosis (white blood cell (WBC) 666 × 10⁹/L, 95% blasts) and severe hyperkalemia (9.4 mmol/L) without electrocardiogram (ECG) changes. Pseudohyperkalemia was considered but ruled out by measuring serum and plasma potassium levels along with using heparinized and non-heparinized tubes. Pseudohyperkalemia is more common in hematologic malignancies due to extreme leukocytosis, which leads to an increase in cell fragility and potassium leakage during sample handling. Despite initial medical therapy, hyperkalemia persisted, requiring emergent hemodialysis, leukapheresis, and cytoreductive treatment. He developed tumor lysis syndrome (TLS), necessitating rasburicase and continuous renal replacement therapy. This case underscores the challenges of hyperkalemia in hematologic malignancies and the importance of rapid differentiation from pseudohyperkalemia.

## Introduction

Hyperkalemia is a potentially life-threatening electrolyte disorder. It may occur in acute kidney injury, metabolic acidosis, and massive cell lysis [1]. In hematologic malignancies, high cell turnover and impaired renal function lead to potassium accumulation, predisposing to cardiac arrhythmias and sudden cardiac death [1]. By contrast, pseudohyperkalemia is a false rise in potassium concentration, a difference of more than 0.4 mmol/L in potassium levels between serum and plasma, due to the lysis of cells during or after blood sample collection, especially in disorders characterized by extreme leukocytosis or thrombocytosis [2,3]. The difference is important because mistakenly interpreting pseudohyperkalemia as true hyperkalemia could result in inappropriate treatment [1]. ECG changes do not always correlate with potassium levels due to the rate of potassium change and individual cardiac variability.

Hyperkalemia acts on cardiac conduction by modifying resting membrane potential, with progressive electrocardiography (ECG) changes [1]. Due to increased permeability to potassium in the myocardial cells, ECG may show peaked T waves, flat P waves, PR interval prolongation, and widening of the QRS complex [1]. Hyperkalemia can cause ventricular fibrillation and cardiac arrest. However, not all ECG changes correlate to potassium levels, hence the need to confirm hyperkalemia via repeat testing [1,3]. Lastly, pseudohyperkalemia is more common in hematologic malignancies due to marked leukocytosis, which leads to an increase in cell fragility and potassium leakage [3]. Distinguishing pseudohyperkalemia from true hyperkalemia is crucial to prevent potentially fatal cardiac arrhythmias [1-3]. This case illustrates the multidisciplinary management of a patient with acute myeloid leukemia who presented with leukocytosis and elevated potassium levels without ECG changes.

## Case presentation

A 66-year-old male presented to the emergency room with worsening, throbbing left upper quadrant abdominal pain radiating to the left shoulder. This pain started a few days ago and got progressively worse. Resting alleviated the pain, while movement exacerbated it. He did not take any medications to relieve his symptoms. The patient denied any injuries to the shoulder or abdominal region, as well as dyspnea, chest pain, nausea, vomiting, diarrhea, or melena. 

He has a past medical history of hypertension; alcohol abuse, resulting in alcoholic hepatitis (abstinence from alcohol for a month); myeloproliferative disorder with positive Janus kinase 2 (JAK2) and isocitrate dehydrogenase 2 (IDH2) mutations; and chronic myelomonocytic leukemia (CMML). He had been taking hydroxyurea months ago and was lost to follow-up. 

On arrival, the patient was hemodynamically stable. Labs were significant for leukocytosis, anemia, thrombocytopenia, hyponatremia, hypernatremia, and elevated uric acid (Table 1).

**Table 1:** Laboratory values on admission

Laboratory	Value	Reference range for our institute
White blood cell count	666	4.5-11.0 (10x3/uL)
Blast cells	95	0 (%)
Hemoglobin	9.3	12.0-16.0 (g/dL)
Platelets	62	140-450 (10x3/uL)
Sodium	126	136-145 (mmol/L)
Potassium	9.4	3.5-5.2 (mmol/L)
Uric acid	11.5	4.0-8.0 (mg/dL)

**Figure 1:**
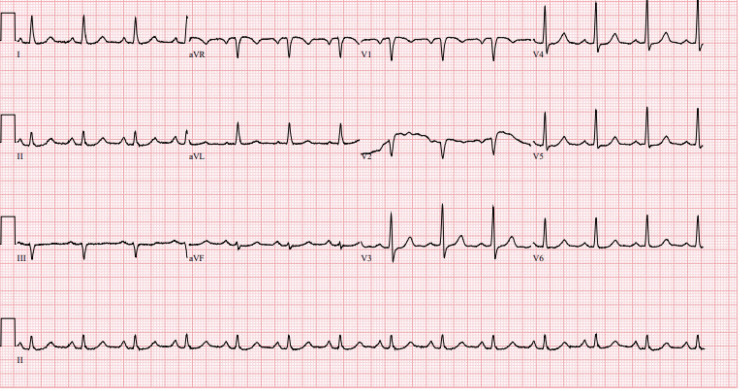
EKG on admission: normal sinus rhythm, possible left atrial enlargement. Ventricular rate: 80 beats per minute. PR interval: 202 millisecond. QRS: 92 millisecond. QTC: 465 millisecond. No peak T-wave changes.

The electrocardiogram (ECG) did not show any peak T-wave or ischemic changes (Figure 1). 

**Figure 2:**
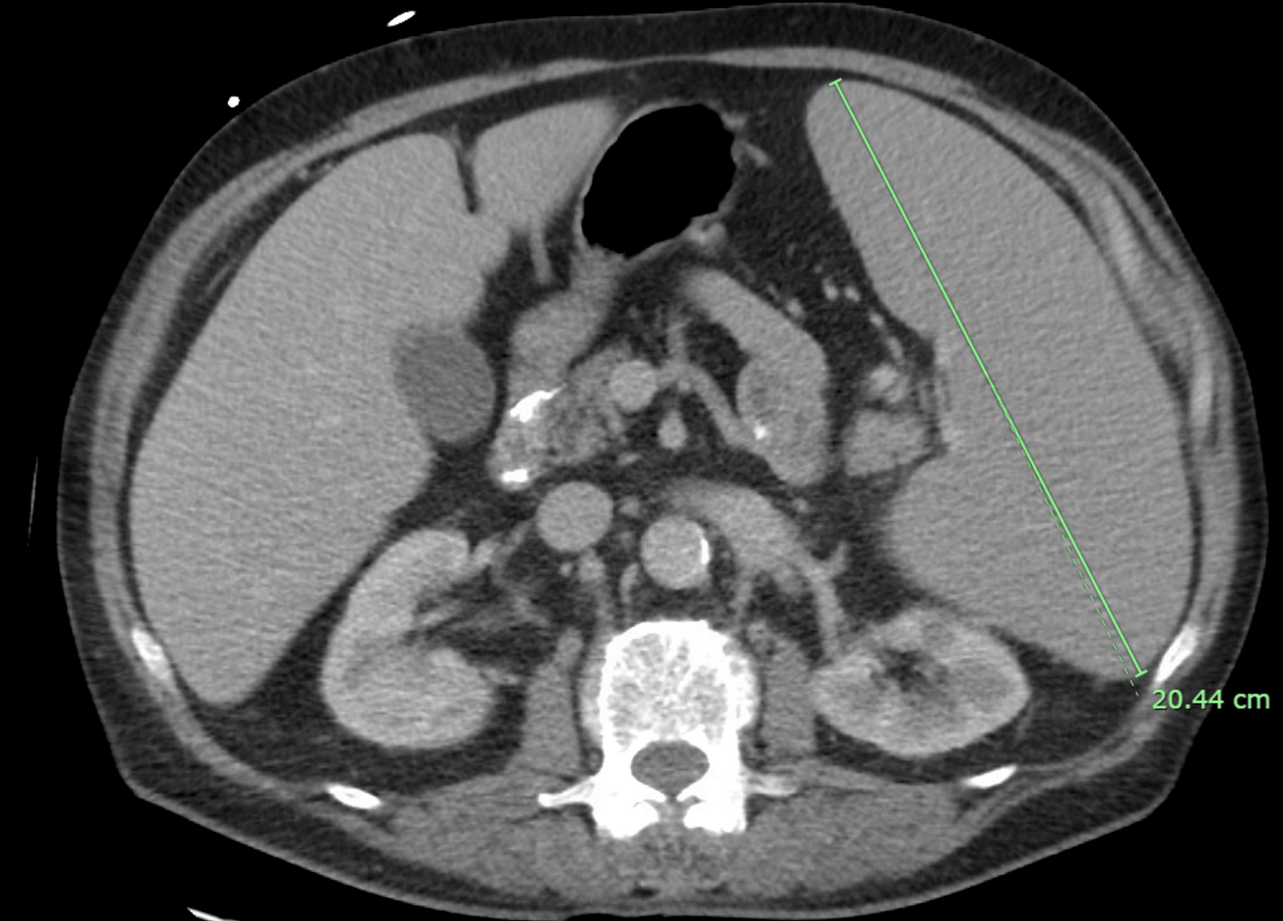
Abdominal imaging remarkable for the enlarged spleen

A computed tomography scan of the chest abdomen and pelvis with contrast showed marked splenomegaly and persistent hepatomegaly (Figure 2).

In the emergency department, the patient received a hyperkalemic cocktail consisting of one amp of sodium bicarbonate, 10 mg sodium zirconium cyclosilicate, 2 g calcium gluconate, eight units of insulin, 40 mg IV furosemide, and 10 mg albuterol nebulizer. He also received 3 grams of rasburicase and started on hydroxyurea 3 grams three times a day. Allopurinol for empiric coverage of tumor lysis syndrome was added. 

**Figure 3:**
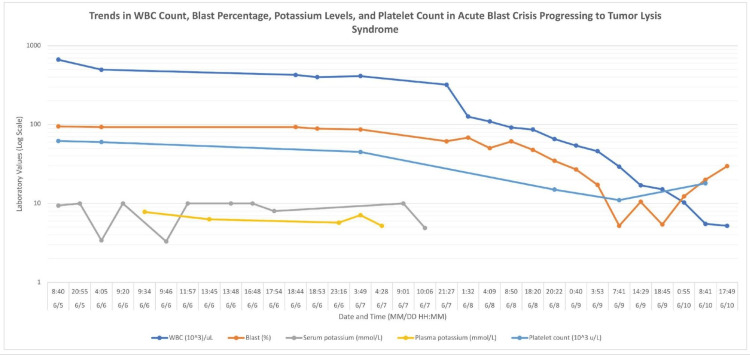
Trends of various laboratory markers showing the progression of acute blast crisis into tumor lysis syndrome

A temporary vascular catheter was placed for the initiation of emergent hemodialysis, and he was started on leukapheresis per the hematology team. Since he had already received one course of therapy aimed at driving potassium intracellularly and given the white cell load and blasts, he had a three-hour hemodialysis treatment, followed by a session of leukapheresis. He was then started on continuous renal replacement therapy (CRRT) after the first dialysis session. The patient's potassium was repeated several times and two separate potassium blood draws were obtained and hand-delivered to the lab to avoid lysis. Given the difficulty in discerning hyperkalemia versus pseudohyperkalemia in the setting of white blood cell fragility in leukemia, his potassium was evaluated in both heparinized and non-heparinized tubes to rule out reverse pseudohyperkalemia [3]. The non-heparinized tube allowed the blood clots to separate before centrifugation, allowing fibrin clots to trap fragile WBC. The heparinized tube in comparison, makes the fragile WBC more susceptible to disruption, sometimes causing pseudohyperkalemia [4]. After the first blood draw (while he was getting hemodialysis), repeat potassium levels for serum and plasma both resulted above 9. Since both plasma and serum potassium were elevated, reverse pseudohyperkalemia had been excluded (Figure 3). 

During the first 24 hours of admission, the repeat potassium after stopping dialysis was 10 after being 3.4 at 0500 and 93% blasts in peripheral blood (Figure 3). 

The patient was eventually transferred to another facility with a leukemia specialist for chemotherapy induction with cytoreductive cytarabine. Throughout follow-up, his potassium levels eventually normalized within a week, and his blast cell crisis improved. He also had clinical improvement in his left upper quadrant abdominal pain.

## Discussion

Potassium (K+) is a vital electrolyte for adequate cellular functioning. Normal potassium levels range from 3.5 to 5.0 mEq/L; both hyperkalemia (potassium >5 mEq/L) and hypokalemia (potassium <3.5 mEq/L) can cause fatal cardiac arrhythmias due to potassium’s key role in cardiac repolarization [5]. 

Pseudohyperkalemia refers to the discrepancy between plasma and serum potassium levels [6]. This is often caused by compromise to the integrity of the cells, such as mechanical stress from excessive centrifugation during lab processing, hemolysis, leukemic cell crisis (as seen in our patient), heparinized versus non-heparinized tubes, temperature fluctuations, or any other form of cellular disruption causing leakage of potassium into the extracellular (serum) space [6]. There are usually no symptomatic or diagnostic manifestations in pseudohyperkalemia compared to hyperkalemia, mainly because pseudohyperkalemia is characterized by only an in vitro (test tube, serum) increase in potassium, without an in vivo (actual intracellular potassium) increase. Hence, pseudohyperkalemia does not require treatment unless the patient is symptomatic, exhibiting accompanying EKG changes, or experiencing hemodynamic compromise [1,7]. 

By contrast, true hyperkalemia is caused by renal dysfunction, tumor lysis syndrome (tumor cells breaking down substances like potassium into the blood too rapidly for the kidneys to clear), medication side effects (e.g., potassium-sparing diuretics), or endocrinological abnormalities [6]. When the correct potassium levels are not accounted for, improper correction of “hyperkalemia” due to pseudo-hyperkalemia can result in dangerously low potassium levels, leading to adverse cardiac events. Likewise, failing to account for true hyperkalemia can also result in adverse events, necessitating proper evaluation of the accurate potassium levels [7]. 

Specifically, leukemic cells pose a risk of pseudohyperkalemia due to their fragile nature, making them more susceptible to lysis from excessive centrifugation, temperature changes, or heparinization [8]. This patient with acute leukemia presented with hyperleukocytosis and numerous circulating blasts, with a WBC of 666,000 and blasts of 95% (Figure 3). His potassium was 9.4 (Table 1), and there were no arrhythmias, signs of ischemia, or significant T-wave changes seen on the EKG (Figure 1). Our lab usually uses pneumatic test tubes for serum chemistry or basic metabolic panels. Pneumatic tubes have rapid acceleration and deceleration in their systems, which can be amplified when not carried upright, ultimately causing increased breakage of cells and increasing the in vivo potassium levels seen in pseudohyperkalemia [8]. In this patient, it was ensured that his test tubes were carried to the lab by hand to prevent falsely elevated potassium levels [9]. The repeat chemistry panels, despite being hand-carried, were still elevated.

Some lab test tubes are coated in heparin because platelets tend to release products into the extracellular space when they are clotting. The anti-clotting effect of heparin prevents this excessive release of products, such as potassium, from platelets. In a study by Garwicz et al. (2012), reverse pseudohyperkalemia done is when there is a high plasma potassium level with a low to normal serum potassium level, which can be explained by the heparinization in the tubes [9]. The heparin-coated tubes prevent the platelets from excessive potassium release during clotting, leading to a relatively low to normal serum potassium level in the extracellular space. Heparin also induces the release of intracellular potassium due to its interactions directly with the cell membrane, leading to a higher level of potassium in the plasma [10]. This was accounted for in our presented patient by running the serial chemistry panels at the same time in a heparinized tube and a non-heparinized tube to assess discrepancies in the potassium levels. Of note, this patient had thrombocytopenia, which would ideally be associated with relatively lower levels of serum potassium. Thrombocytosis (increased platelets) is associated with pseudohyperkalemia due to the overall increase in platelet release of potassium during clotting [10,11]. Reverse pseudohyperkalemia was ruled out in this case, as the repeat potassium levels remained high in both heparinized and non-heparinized tubes.

The incidence of blast cell crisis in patients with acute myeloid leukemia is rather common; however, there is a risk of an underreported number of these cases due to the overlooking of pseudohyperkalemia versus hyperkalemia [10]. This demonstrates the importance of proper diagnostic evaluations in these populations. In our patient, serial chemistry panels were done under specialized circumstances to ensure the potassium values were as accurate as possible. His tubes were hand-carried to prevent mechanical stress to the leukemic cells, and pneumatic tubes were accounted for by communicating with the lab personnel to prevent unnecessary centrifugation of these samples. Likewise, the chemistry was run simultaneously on one heparinized tube and one non-heparinized tube, both showing elevated serum potassium levels at the same time. These findings demonstrated that, despite his lack of EKG changes, the patient had truly high potassium levels in the blood rather than pseudohyperkalemia.

**Figure 4:**
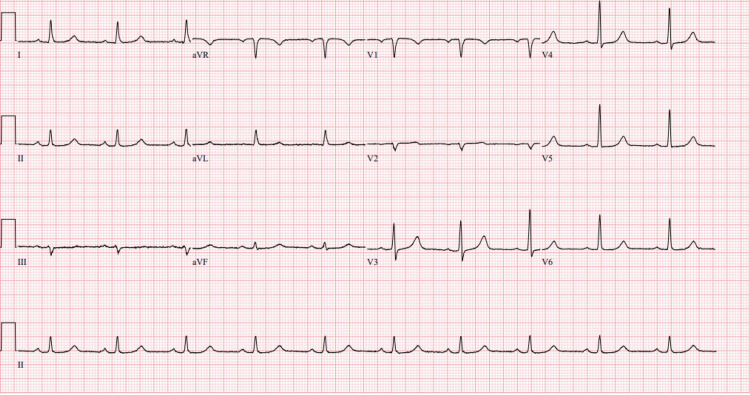
EKG after improvement of his blast cell crisis and hyperkalemia-normal sinus rhythm, no ischemic or peak T-wave changes. Ventricular rate: 61 beats per minute. PR interval: 198 millisecond. QTc: 469 millisecond.

**Figure 5:**
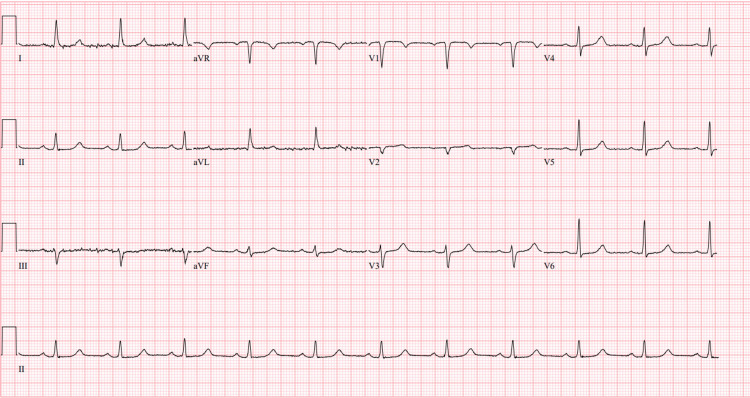
No ischemic changes noted. Negative for peak T wave. Ventricular rate: 64  beats per minute. PR interval: 198 millisecond. QTc: 474  millisecond.

To prevent any risk of true hyperkalemia from tumor lysis syndrome in his setting of cancer (his uric acid was around 11), he started on fluids and rasburicase. He was also given 3 g IV calcium gluconate to empirically prevent fatal arrhythmias if there was true hyperkalemia. Once true causes of hyperkalemia are addressed in a timely fashion, it is equally crucial to acknowledge the role of pseudohyperkalemia and to caution against the dangers of over-correcting electrolytes and risking arrhythmias. This patient underwent continuous renal replacement therapy urgently, as well as a few rounds of leukapheresis until his blast cell crisis decreased over the next few weeks. The ICU, hematology/oncology, and nephrology teams approached his treatment in a multidisciplinary manner. They ensured that the leukapheresis, CRRT, and any electrolyte repletion were done in coordination with other team members, given the delicate nature of his lab values. Serial ECGs were done to monitor for life-threatening arrhythmias or T wave aberrancies (Figures 4, 5). 

Caution in management

It is imperative to reiterate the meticulous handing of blood samples in leukemic patients. In vitro, factors such as sample collection and transport can cause mechanical stress leading to abnormal levels [12,13]. Failure to promptly recognize the clinical scenario can lead to cardiac complications because elevated potassium levels directly affect myocardial activity resulting in arrhythmias and cardiac arrest [14]. Although standard medical treatments seek to reduce potassium levels, this patient’s state requires more aggressive measures. Leukapheresis was performed before administering induction chemotherapy so that the very high white blood cells could be lowered to mitigate the risk of TLS [15]. Later, CRRT was started to treat renal failure. Aggressive cytoreduction successfully controlled hyperleukocytosis; however, this had to be balanced against the risk of tumor lysis syndrome. Over the course of the treatment, the patient's abdominal pain improved, likely due to a reduction in spleen size, which was enlarged from leukemic infiltration. Reduction in spleen size alleviated associated pressure, leading to symptom relief. Ultimately, the knowledge of pseudohyperkalemia, combined with a collaborative team approach, led to the patient’s clinical improvement in a few weeks.

## Conclusions

This case highlights the importance of differentiating between pseudohyperkalemia and hyperkalemia in the setting of a blast cell crisis in leukemia. Given potassium’s crucial role in maintaining heart function and organ health, it is essential to develop the diagnostic skills to distinguish between pseudohyperkalemia and true hyperkalemia, as well as to know how to treat them. In this patient, after extensive testing ruled out pseudohyperkalemia, he was found to have true hyperkalemia, which responded to treatment with CRRT and leukapheresis. The risk of aggressive cytoreduction must be balanced against the risk of developing TLS. Eventually, his potassium levels and uric acid normalized, and his initial symptoms of upper abdominal pain slowly improved. In potassium derangement, cautious monitoring of subsequent lab values and investigation of the etiology is vital in preventing life-threatening arrhythmias in these patients.
